# MASP-1 Increases Endothelial Permeability

**DOI:** 10.3389/fimmu.2019.00991

**Published:** 2019-05-03

**Authors:** Márta L. Debreczeni, Zsuzsanna Németh, Erika Kajdácsi, Endre Schwaner, Veronika Makó, András Masszi, Zoltán Doleschall, János Rigó, Fruzsina R. Walter, Mária A. Deli, Gábor Pál, József Dobó, Péter Gál, László Cervenak

**Affiliations:** ^1^Research Laboratory, 3rd Department of Internal Medicine, Semmelweis University, Budapest, Hungary; ^2^MTA-SE Research Group of Immunology and Hematology, Hungarian Academy of Sciences and Semmelweis University, Budapest, Hungary; ^3^Department of Pathogenetics, National Institute of Oncology, Budapest, Hungary; ^4^First Department of Obstetrics and Gynecology, Semmelweis University, Budapest, Hungary; ^5^Biological Research Centre, Institute of Biophysics, Hungarian Academy of Sciences, Szeged, Hungary; ^6^Department of Biochemistry, Eötvös Loránd University, Budapest, Hungary; ^7^Research Centre for Natural Sciences, Institute of Enzymology, Hungarian Academy of Sciences, Budapest, Hungary

**Keywords:** MASP-1, endothelial cell, permeability, PAR-1, angioedema, C1-inhibitor, XPerT assay, transcriptome analysis

## Abstract

Pathologically increased vascular permeability is an important dysfunction in the pathomechanism of life-threatening conditions, such as sepsis, ischemia/reperfusion, or hereditary angioedema (HAE), diseases accompanied by uncontrolled activation of the complement system. HAE for example is caused by the deficiency of C1-inhibitor (the main regulator of early complement activation), which leads to edematous attacks threatening with circulatory collapse. We have previously reported that endothelial cells become activated during HAE attacks. A natural target of C1-inhibitor is mannan-binding lectin-associated serine protease-1 (MASP-1), a multifunctional serine protease, which plays a key role in the activation of complement lectin pathway. We have previously shown that MASP-1 induces the pro-inflammatory activation of endothelial cells and in this study we investigated whether MASP-1 can directly affect endothelial permeability. All experiments were performed on human umbilical vein endothelial cells (HUVECs). Real-time micro electric sensing revealed that MASP-1 decreases the impedance of HUVEC monolayers and in a recently developed permeability test (XperT), MASP-1 dose-dependently increased endothelial paracellular transport. We show that protease activated receptor-1 mediated intracellular Ca^2+^-mobilization, Rho-kinase activation dependent myosin light chain (MLC) phosphorylation, cytoskeletal actin rearrangement, and disruption of interendothelial junctions are underlying this phenomenon. Furthermore, in a whole-transcriptome microarray analysis MASP-1 significantly changed the expression of 25 permeability-related genes in HUVECs—for example it up-regulated bradykinin B2 receptor expression. According to our results, MASP-1 has potent permeability increasing effects. During infections or injuries MASP-1 may help eliminate the microbes and/or tissue debris by enhancing the extravasation of soluble and cellular components of the immune system, however, it may also play a role in the pathomechanism of diseases, where edema formation and complement lectin pathway activation are simultaneously present. Our findings also raise the possibility that MASP-1 may be a promising target of anti-edema drug development.

## Introduction

Mannan-binding lectin-associated serine protease-1 (MASP-1) is a multifunctional serine protease of complement and coagulation. MASP-1 is indispensable for the activation of the complement lectin pathway, circulating in a zymogen form in complex with pattern recognition molecules (PRMs) mannan binding lectin (MBL), ficolins or collectins, serin proteases MASP-2 and MASP-3, and non-catalytic proteins MAp19/MAP-2 and Map44/MAP-1 (1, 2). The most well-known function of MASP-1 is its central, zymogen MASP-activating role during lectin pathway activation (3, 4). According to the latest model, MASP-1 auto-activates upon the binding of the PRM to an activator surface, then activates zymogen MASP-2, which then cleaves C4, and both MASP-1 and MASP-2 cleave C2 to form C3 convertase C4bC2a (2). Although, being a promiscuous protease with a relatively large and open substrate-binding cleft (5), MASP-1 is capable of cleaving a great variety of substrates in- and outside the complement system. It can activate MASP-3 and possibly contribute to alternative pathway activation (4, 6). Furthermore, it cleaves fibrinogen, Factor XIII, thrombin activatable fibrinolysis inhibitor (TAFI) and prothrombin to promote blood clotting and coagulation (7–10). MASP-1 is also able to deliberate the potent edematogenic agent bradykinin from high molecular weight kininogen (HMWK) (11). Moreover, as we have shown previously, it can activate endothelial cells (ECs) (12, 13), triggering the production of interleukins IL-6 and IL-8 (14) and up-regulating endothelial E-selectin expression (15).

Activity of MASP-1 is controlled from both in- and outside the initiation complex. An intracomplex inhibitor of MASP activity is the above mentioned MAP-1 that was found to be able to displace all three MASPs on MBL. In mouse models, administration of MAP-1 successfully preserved cardiac function, decreased infarct size and C3 deposition, inhibited MBL deposition and prevented thrombogenesis (1). Serine protease activity of MASP-1, however, is controlled mainly by C1-inhibitor (C1-INH), a circulating serine protease inhibitor, which is the most important regulator of early complement activation and several other plasma enzyme systems. Genetic deficiency of C1-INH causes hereditary angioedema (HAE), a rare, life-threatening disease characterized by vascular hyperpermeability leading to edematous attacks (16, 17). We have also shown that during these attacks, ECs become activated (18).

Since C1-INH is not specific for MASP-1, we have developed a highly specific, artificial MASP-1 inhibitor, SGMI-1 (*Schistocerca gregaria* protease inhibitor-based MASP-1 inhibitor) to study the physiological and pathophysiological roles of MASP-1 (19).

ECs have numerous physiological roles; besides regulating blood-pressure, hemostasis, leukocyte homing, and several other processes, they control vascular permeability, which can take place via both paracellular and transcellular routes. The well-known permeability inducing agonists—such as thrombin, histamine, or bradykinin—increase the intensity of paracellular transport by disrupting endothelial cell-cell adhesion (20, 21).

Thrombin exerts its cellular effects by cleaving protease activated receptors (PARs) on the surface of ECs. These receptors are members of the G-protein coupled receptor family. PARs have an N-terminal region sensitive to proteolytic cleavage, and specific limited proteolysis within this segment unmasks a tethered, self-activating ligand on the receptor. This protease-induced receptor activation elicits a rise in intracellular [Ca^2+^], and through a complex signaling mechanism involving myosin light chain kinase (MLCK) and Rho-kinase (ROCK) activation leads to myosin light chain (MLC) di-phosphorylation, actin stress fiber formation, cell contraction, redistribution of junctional adhesion- and adapter molecules—for example vascular endothelial cadherin (VE-cadherin) and zonula occludens-1 (ZO-1)—and finally, dissociation of endothelial cell-cell junctions (22). MASP-1 and thrombin share several structural and functional similarities (5) and we have previously reported that similarly to thrombin, MASP-1 can also activate ECs by cleaving cell surface PARs (12, 13). It is also known that in the pathomechanism of HAE, deficiency of C1-INH—the natural inhibitor of MASP-1—leads to edema formation due to increased vascular permeability. Furthermore, in certain cases—such as bacterial/fungal infection, tissue necrosis, or inflammation triggered HAE attacks—lectin pathway was found to be activated during attacks (23). These observations led us to ask the question, whether MASP-1 can directly affect endothelial permeability.

Here we show that the recombinant catalytic fragment of MASP-1 (rMASP-1), which is enzymatically equivalent with full-length plasma purified MASP-1, significantly and dose-dependently increases endothelial permeability in a PAR-1 and ROCK dependent manner. Furthermore, we demonstrate that rMASP-1 affects the expression of permeability-related genes in human umbilical vein ECs (HUVECs) as well.

## Materials and Methods

### Reagents

rMASP-1 was produced in *Escherichia coli* (24) and purified by the method described by Dobó et al. (25). Purity of rMASP-1 preparations was checked as described earlier (13, 15) and found to be free of bacterial contaminations. Furthermore, C1-INH completely inhibited all tested effects of rMASP-1, and the enzymatically inactive mutant (S646A) as well as the zymogen mutant (R448Q) variants of rMASP-1 (produced in the same expression system (4) did not elicit any cellular response.

The highly selective MASP-1 inhibitor SGMI-1 used in our experiments was produced according to the method described earlier by Héja et al. (19), and was found to be non-toxic to ECs.

Other reagents were purchased from Sigma-Aldrich, unless stated otherwise.

### Preparation and Culturing of HUVECs

ECs were harvested by collagenase digestion from fresh umbilical cords obtained during normal deliveries of healthy neonates as described earlier (14, 26). HUVECs were cultured in gelatin pre-coated flasks (Corning® Costar®) in AIM-V medium (Life Technologies) completed with 1% filtrated, heat inactivated bovine serum (PAN Biotech), 1 ng/mL human recombinant basic fibroblast growth factor, 2 ng/mL human recombinant epidermal growth factor (R&D Systems), and 7.5 U/mL heparin; hereinafter referred to as Comp-AIM-V.

Experiments were performed on HUVECs from at least three different individuals, before passage 4. The study was conducted in conformity with the WMA Declaration of Helsinki; its protocol was approved by the Semmelweis University Institutional Review Board (permission number: TUKEB141/2015). All participants provided their written informed consent before inclusion.

### Real-Time Cell Microelectronic Sensing

Changes in the impedance of HUVEC monolayers were measured using the xCELLingence system (Roche, Hungary) as described earlier (27). Briefly, a 96-well E-plate (Roche, Hungary) mounted by golden microelectronic sensor arrays was pre-coated with 0.5% gelatin, and HUVECs were seeded onto the surface and cultured for 3 days. Confluent HUVEC monolayers were treated with (i) 2 μM rMASP-1; or (ii) 100 nM thrombin or (iii) culture medium. Throughout the experiments, cells were kept in incubator at 37°C and monitored every 1 min. Cell index for each time point was determined using the following formula: (Rn—Rb)/15, where Rn is the cell-electrode impedance of the well when it contains cells and Rb is the background impedance of the well with the medium alone.

### Permeability Measurements

Permeability tests were carried out using a modified version of the recently developed XPerT technique (28). Confluent layers of HUVECs were seeded onto 96-well plates pre-coated with 250 μg/ml biotinylated gelatin, and were cultured in Comp-AIMV medium for 2 days. After cell treatment, Streptavidin-Alexa488 (2 μg/ml, Life Technologies) was added to each well for 2 min. Cells were fixed with 1% paraformaldehyde-PBS and 2 pictures of each well were taken using an Olympus IX-81 fluorescence microscope and an Olympus XM-10 camera. Size of the stained area was determined on each image by quantitative image analysis using the CellP software (Olympus). Thrombin (300 nM, Merck Millipore) was used as a positive control in every test.

Dose dependence of rMASP-1 treatment was examined using 0.2, 0.6, or 2 μM of rMASP-1. In other experiments rMASP-1 was used at 2 μM. Inhibitors of rMASP-1, 6 μM of human plasma derived C1-INH (Berinert®, CSL Behring) or 20 μM of SGMI-1 were pre-incubated with rMASP-1 at room temperature for 30 min before treating HUVECs. In experiments investigating the PAR-dependence of the rMASP-1 triggered permeability, cells were either treated with one of the following PAR antagonists, PAR-1: RWJ 56110; PAR-2: FSLLRY-NH_2_; PAR-4: ML 354 for 5 min, or with the culture medium alone, and then were treated either with rMASP-1 or one of the following PAR agonists, PAR-1: TFLLR-NH_2_; PAR-2: AC 55541; PAR-4: AY-NH_2_ for 20 min—for the concentrations used, see **Figure 3**. All antagonists and agonists were purchased from Tocris Bioscience. Effects of the inhibition of protein kinases MLCK and ROCK on rMASP-1 induced permeability was tested by pretreating the cells with inhibitors ML-7 (MLCK inhibitor, 10 μM, Tocris Bioscience) and Y-27632 (ROCK inhibitor, 2.5 μM, Tocris Bioscience) for 15 min before rMASP-1 treatment. Minimum doses of PAR-agonists/-antagonists and protein kinase inhibitors ML-7 and Y-27632 were determined according to the literature data.

### Intracellular Ca^2+^ Mobilization Assay

Intracellular Ca^2+^ mobilization has been measured as described earlier (12). Confluent layers of HUVECs were seeded onto 96-well plates and cultured in Comp-AIM-V for 1 day. Cells were loaded with 2 μM Fluo-4-AM (Molecular Probes) for 20 min, then incubated in HBSS for another 20 min. Cells were treated with or without PAR antagonists for 5 min, then sequential images were obtained every 5 s by fluorescence microscopy. Three photos were taken to determine the baseline fluorescence, then cells were treated with 2 μM rMASP-1 or PAR-agonists and the response was measured for 2 min. (For PAR-agonist and—antagonist concentrations, see **Figure 4**.)

Twenty cells per image were then analyzed using the CellP software.

### MLC Phosphorylation and Cytoskeletal Actin Visualization

Confluent layers of HUVECs were seeded onto 18-well ibidi™ slides and cultured in Comp-AIMV medium for 2 days. Cells were pretreated with or without PAR-antagonists for 5 min, then were treated either with 2 μM rMASP-1 or with 300 nM thrombin for 20 min. In other experiments, cells were pretreated with 10 μM ML-7 or 2.5 μM Y-27632 or culture medium only for 15 min. After 1% paraformaldehyde-PBS fixation, cells were stained with anti-diphospho MLC (pMLC) antibody (Cell Signaling). F-actin cytoskeleton was stained with phalloidin-Alexa488. Cell nuclei were labeled with Hoechst 33258 (Thermo Fisher, 200 ng/mL). Pictures were taken using a fluorescence microscope and camera described above. For the calculation of co-localization (Pearson's *R* value), we used ImageJ 1.52i software, Coloc2 analysis (Costes threshold regression) on background subtracted, deconvoluted pictures.

### Visualization of Adhesion Molecules

Confluent layers of HUVECs were seeded onto 18-well ibidi^TM^ slides and cultured for 2 days. Cells were treated with (i) 300 nM thrombin; or (ii) 2 μM rMASP-1; or (iii) a mixture of 2 μM rMASP-1 and 6 μMC1-INH; or (iv) culture medium for 20 min. After fixation, cells were stained with anti-VE-cadherin (Santa Cruz) anti-ZO-1 (Invitrogen) or anti-platelet-endothelial cell adhesion molecule-1 (PECAM-1) (Bender MedSystems) antibodies, followed by Alexa-488 labeled secondary antibodies. Cell nuclei were labeled with Hoechst 33258. Images were taken using fluorescence microscopy.

### Whole Transcriptome Microarray Analysis

Total RNA isolation and microarray processing were carried out as described in our earlier study (29). Briefly, confluent layers of HUVECs from four individuals were cultured in 6-well plates and treated for 2 h with 0.6 μM rMASP-1. To compare the effects of rMASP-1 with other EC activators, cells were treated with one of the following compounds: 300 nM thrombin, 10 ng/mL tumor necrosis factor-α (TNFα), 100 ng/mL lipopolysaccharide (LPS), 50 μM histamine. All microarray data are available at the Gene Expression Omnibus database at NCBI (series accession number GSE98114). Microarray data were validated by quantitative polymerase chain reaction for 19 genes, 3 of which—F3, TGFBR1 and EDNRB—were permeability-related genes.

From the set of genes having expression levels significantly altered by rMASP-1 treatment, genes without known connection with permeability were excluded based on REACTOME, KEGG and GO databases. Experimental evidence for the permeability related function of the remaining list of genes was verified according to the current literature. Genes that could be linked to permeability regulation were divided into three groups based on the type of evidence for their role in the modulation of endothelial permeability: (i) direct evidence—protein products of these genes *have been directly proven* to affect endothelial permeability (ii) indirect evidence—protein products of these genes have been proven to affect the function of known permeability regulating factors, and (iii) potential role—protein products of these genes are suspected to influence the barrier properties of the endothelium. Genes in the (i) and (ii) categories are also proven to be expressed in ECs at protein level. Table 1 contains the median fold-change (FC) values of 4 independent HUVECs.

**Table 1:** rMASP-1 significantly changes the expression of permeability related genes.

**Median of fold change by rMASP-1**	**Gene symbol**	**Gene name by HUGO Gene Nomenclature Committee (HGNC)**	**Description of the permeability related function**	**Other activators**			
				**LPS**	**Histamine**	**Thrombin**	**TNF-α**
**UP-REGULATED**							
4.09	F3	Coagulation factor III, tissue factor	Important mediator of the endothelial hyperpermeability induced by TNF-α, which exerts its permeability increasing effect by the up-regulation of endothelial cell surface tissue factor (30)	↑	↑	↑	↑
3.38	FZD7	Frizzled class receptor 7	Adherent junction (AJ) protein which co-localizes with VE-cadherin and has an important role in the stabilization of the endothelial barrier (31)				
3.35	RCAN1	Regulator of calcineurin 1	Synthetized in response to histamine treatment, and has a role in reducing endothelial barrier breakdown (32)	↑	↑	↑	↑
2.78	GHSR	Growth hormone secretagogue receptor	Mediates the effect of ghrelin. Reduces the permeability increasing effect of LPS (33) and also prevents the increase in the blood-brain barrier permeability following traumatic brain injury (34)	↑			
2.54	FOXF1	Fork head box F1	Transcription factor important in the maintenance of endothelial barrier function (35)	↑	↑	↑	↑
2.32	TLR2	Toll like receptor 2	Mediates post-ischemic permeability increase (36)	↑			
2.24	KITLG	KIT ligand	Binds to endothelial c-Kit receptor and increases endothelial permeability through the stimulation of VE-cadherin internalization (37, 38)	↑	↑		
2.17	BDKRB2	Bradykinin receptor B2	Receptor for the well-known edematogenic factor bradykinin. Also an important pharmacological target in HAE therapy (39)			↑	↑
2.03	TGFBR1	Transforming growth factor beta receptor 1	TGF-β induces endothelial cell contraction and increases endothelial permeability through this receptor (40, 41)	↑	↑	↑	
**DOWN-REGULATED**							
−2.90	EDNRB	Endothelin receptor type B	Receptor for endothelin-1, which reduces the permeability increasing effect of bradykinin and ATP through this receptor (42)	↓	↓	↓	↓
−2.58	APLNR	Apelin receptor	Mediator of the effects of apelin, which is important for the stabilization of the endothelial barrier of both blood and lymphatic vessels (43–45)				
−2.30	CCR3	C-C motif chemokine receptor 3	Eotaxin increases endothelial permeability through this receptor (46)	↓			

*Confluent layers of HUVECs from four individuals were cultured in 6 well plates and treated for 2 h with 0.6 μM rMASP-1. To compare the effects of rMASP-1 with other endothelial cell (EC) activators, cells were treated with 300 nM thrombin, 10 ng/mL TNFα, 100 ng/mL LPS, or 50 μM histamine. The table contains the median fold-change (FC) values of the 4 independent HUVECs. From the set of genes significantly changed by rMASP-1, possible permeability-related genes were filtered out using REACTOME, KEGG, and GO databases. Experimental evidence for the permeability related function of these genes were verified according to the current literature. This table contains only those genes with a protein product proven to play a role in endothelial permeability regulation supported by direct experimental evidence. Terms and pathways used to filter out possible permeability related genes from databases: GO—permeability; junction; actin; adhesion; camp; cgmp; rho; calcium. REACTOME—Cell junction organization; Extracellular matrix organization; Cell surface interactions at the vascular wall; Toll-like receptors cascades; Complement cascade; Nucleotide-binding domain, leucine rich repeat containing receptor (NLR) signaling pathways. KEGG—Cell adhesion molecules; Adherens junction; Focal adhesion; Tight junction; Gap junction; Complement and coagulation cascades; Toll-like receptor signaling pathway; NOD-like receptor signaling pathway; RIG-I-like receptor signaling pathway; Leukocyte transendothelial migration. **↑**: Gene expression was up-regulated by the given activator; **↓**: Gene expression was up-regulated by the given activator*.

### Statistical Analysis

Experiments were performed in triplicates and repeated at least three times using HUVECs from different individuals. Statistical analysis was done using the Prism 5.01 software (GraphPad). One-way ANOVA with Tukey post test was conducted to determine the statistical significance of the results. A *p* ≤ 0.05 was considered significant.

## Results

### rMASP-1 Treatment Decreases the Impedance of Endothelial Monolayers

**Figure 1:**
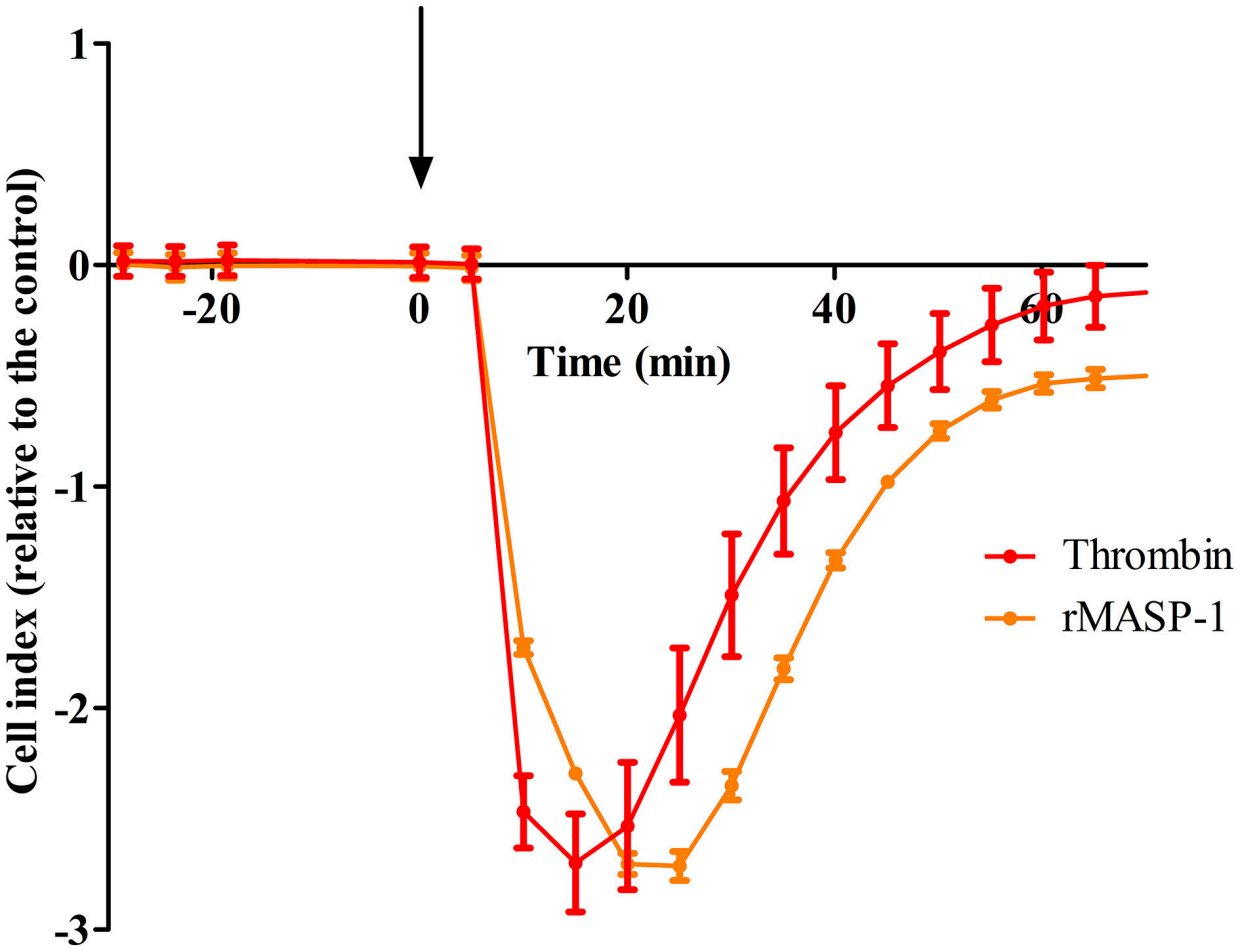
rMASP-1 treatment reversibly decreases the impedance of the endothelial monolayer. 96 well E-plate (Roche, Hungary) mounted by golden microelectronic sensor arrays was pre-coated with 0.5% gelatin, and HUVECs were seeded onto the surface and cultured for 3 days. Confluent HUVEC monolayers were treated with 2 μM rMASP-1 or 100 nM thrombin or the culture medium only. Throughout the experiments, cells were kept in incubator at 37°C and monitored every 1 min. Cell index for each time point was determined using the following formula: (Rn—Rb)/15, where Rn is the cell-electrode impedance of the well when it contains cells and Rb is the background impedance of the well with the medium alone. Values of cell index are therefore dimensionless and are directly proportional to the electrical permeability of the cell layer. Cell indices of MASP-1 and thrombin treated cells were normalized with that of the control (cells treated with growth medium only). Representative of three independent experiments. The arrow indicates the addition of treatment.

To explore whether MASP-1 has any effect on the barrier function of endothelial monolayers, we conducted real-time impedance measurements using the xCELLigence system. Treating HUVECs with 2 μM rMASP-1 resulted in a sharp decline of impedance with a minimum at 15–20 min after treatment and an overall duration of approximately 1 h, around which impedance has stabilized at a level slightly under baseline (Figure 1). This effect of rMASP-1 was similar to the response triggered by the well-known permeability increasing agonist thrombin.

### rMASP-1 Significantly and Dose-Dependently Increases Endothelial Permeability

Although results with the xCELLigence system postulated that MASP-1 can increase endothelial permeability, the findings needed to be confirmed, as xCELLigence does not measure true mass transport. Permeability tests were carried out using a modified version of the recently developed XPerT technique, which is a simple, high-throughput and cost-effective assay for the measurement of true mass transport through endothelial monolayers.

**Figure 2:**
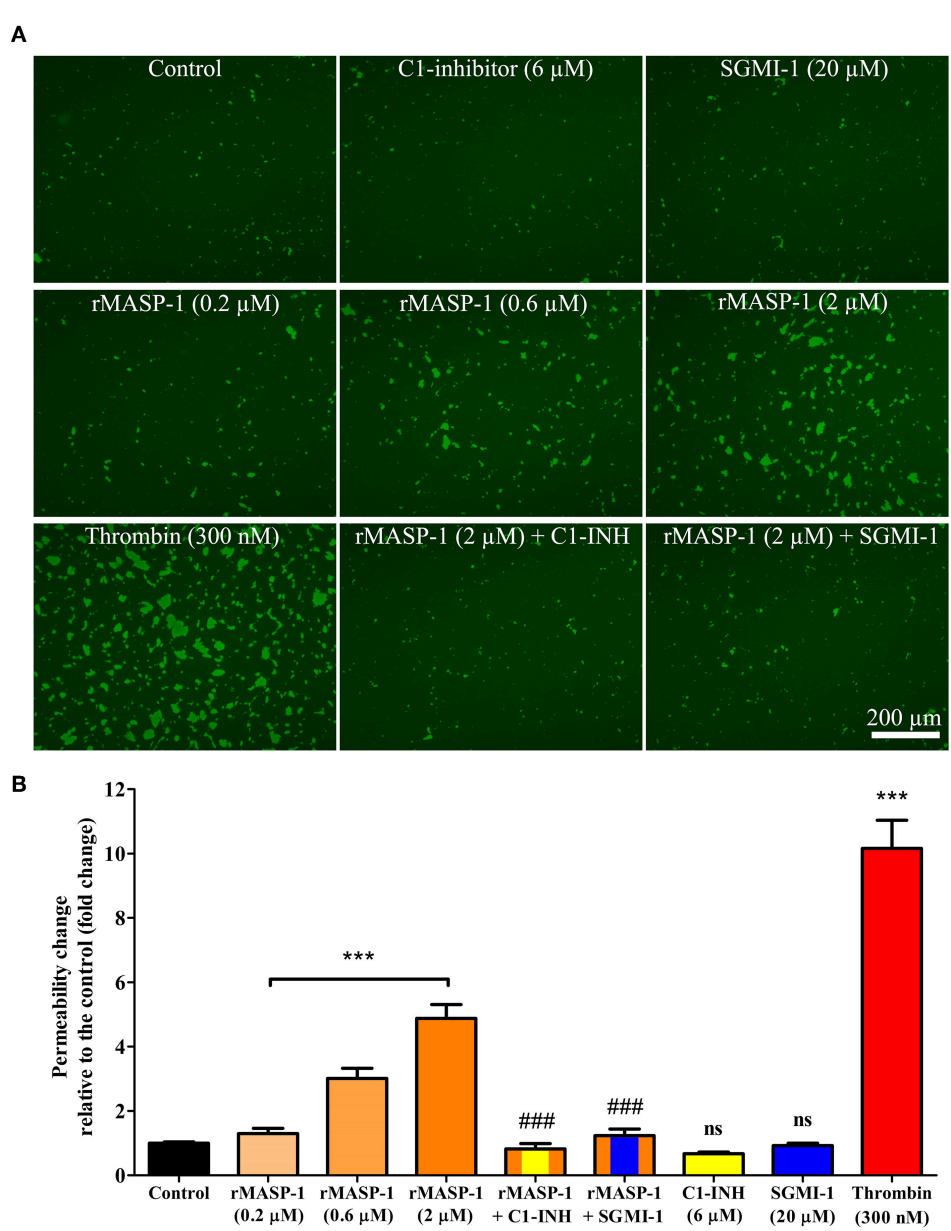
rMASP-1 treatment increases endothelial permeability. Confluent layers of HUVECs were seeded onto 96 well plates pre-coated with biotinylated gelatin and were treated with various concentrations of rMASP-1 or a mixture of rMASP-1 and its inhibitors SGMI-1 or C1-inhibitor together or with the culture medium alone (control) for 20 min. Streptavidin-Alexa488 was added to each well, and after cell fixation, pictures were taken using an Olympus IX-81 fluorescence microscope and an Olympus XM-10 camera with 10x magnification Olympus lenses (numerical aperture: 0.3). **(A)** Representative images of three independent experiments. The scale bar applies to all photomicrographs. **(B)** Size of the stained area was determined on each image using the CellP software. Mean values of three independent experiments normalized to the controls are shown. ***p < 0.005, compared to the control, analyzed by one-way ANOVA and post-test for linear trend; ns, non-significant; ###*p* < 0.005, compared to the 2 μM rMASP-1 treated.

rMASP-1 induced an approximately 5-fold increase in the permeability of HUVEC monolayers, and thrombin, used as positive control, triggered a comparable permeability change (Figure 2). rMASP-1 elicited permeability was dose dependent and was completely inhibited by C1-INH and also by SGMI-1.

### rMASP-1 Induced Endothelial Permeability Is Dependent on PAR-1 and ROCK Activation

**Figure 3:**
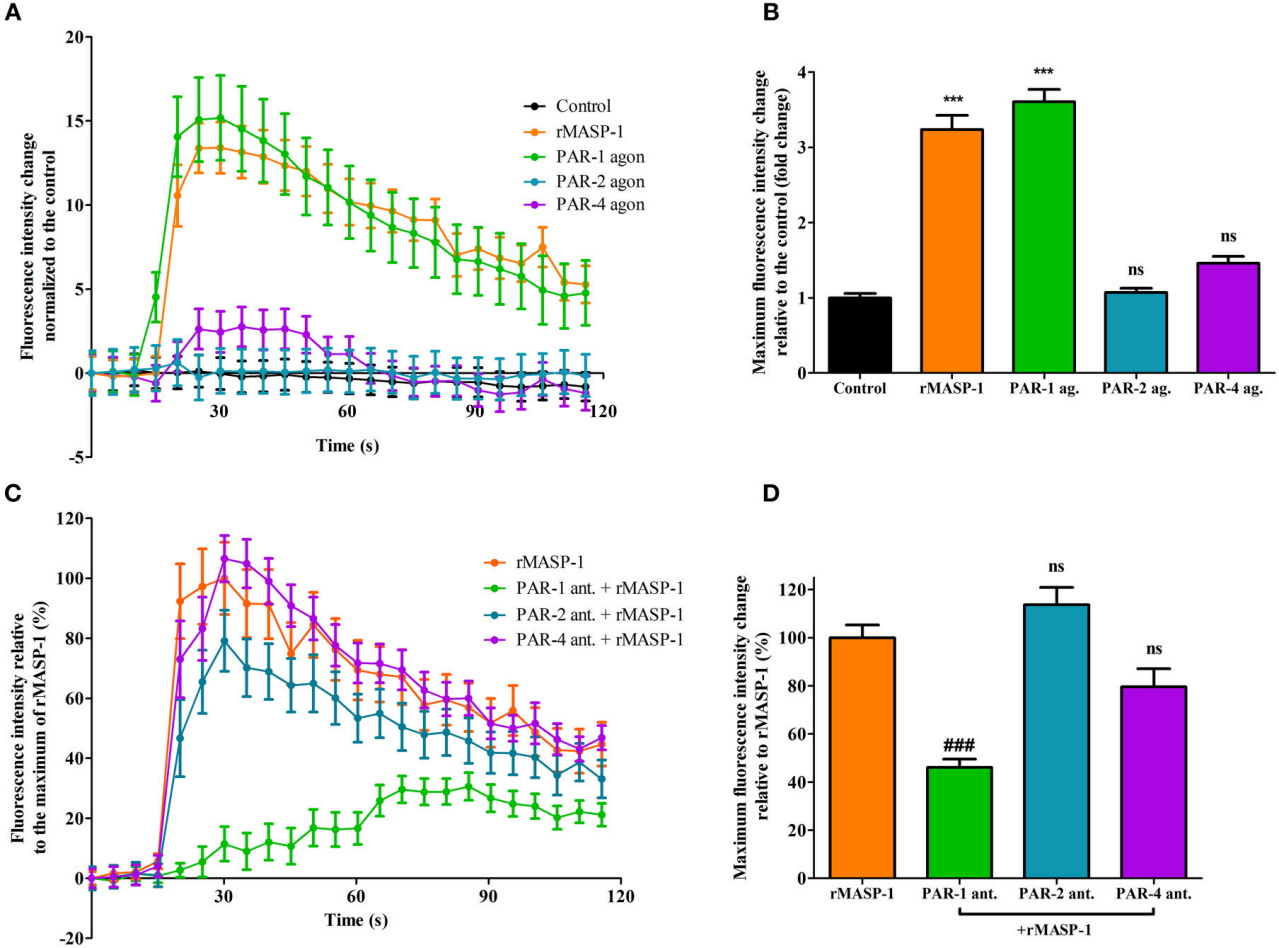
PAR-1 mediates the rMASP-1 triggered intracellular Ca^2+^ mobilization in HUVECs. Confluent layers of HUVECs were seeded onto 96 well plates and cultured for 1 day. Cells were loaded with Fluo-4 AM. **(A,B)** Sequential images were obtained every 5 s by fluorescence microscopy using an Olympus IX-81 fluorescence microscope and an Olympus XM-10 camera with 20x magnification Olympus lenses (numerical aperture: 0.45). Three photos were taken to determine the baseline fluorescence, then cells were treated with 2 μM rMASP-1 or PAR-agonists (4 μM PAR-1 agonist, 0.4 μM PAR-2 agonist or 1 mM PAR-4 agonist) or the culture medium only (control). **(C,D)** Cells were pretreated with or without PAR antagonists (0.68 μM PAR-1 antagonist; 20 μM PAR-2 antagonist or 0.28 μM PAR-4 antagonist) for 10 min. Sequential images were obtained every 5 s by fluorescence microscopy. Three photos were taken to determine the baseline fluorescence, then cells were treated with rMASP-1 or with culture medium alone (control) and the response was measured for 2 min. Images were then analyzed using the CellP software. **(A,C)** data from a single, representative experiment, where fluorescence intensity values were background corrected and normalized to the control. **(B)** Means of the maximum fluorescence intensity values normalized to that of the control are presented. Data from three independent experiments. **(D)** Means of the maximum fluorescence intensity values are expressed as the percentage of rMASP-1 treatment (control: 0%). Data from three independent experiments. ***p < 0.005, compared to the control; ###*p* < 0.005 compared to the rMASP-1 treated; ns, non-significant.

We previously reported that rMASP-1 can cleave PAR-1, PAR-2, and PAR-4. Since PAR-dependent cell activation accompanied by intracellular Ca^2+^ mobilization is an important step in permeability induction, we investigated the role of various PARs in the rMASP-1 triggered Ca^2+^ response in HUVECs using PAR-agonists and -antagonists. Treating the cells with PAR-1 agonist induced a strong increase of intracellular [Ca^2+^], while the PAR-4 agonist only had a mild effect. The PAR-2 agonist failed to elicit any response (Figures 3A,B). In accordance with these results, rMASP-1 triggered Ca^2+^ mobilization was significantly blocked by PAR-1 antagonist pretreatment, while PAR-2 and PAR-4 antagonists showed no significant effect (Figures 3C,D).

**Figure 4:**
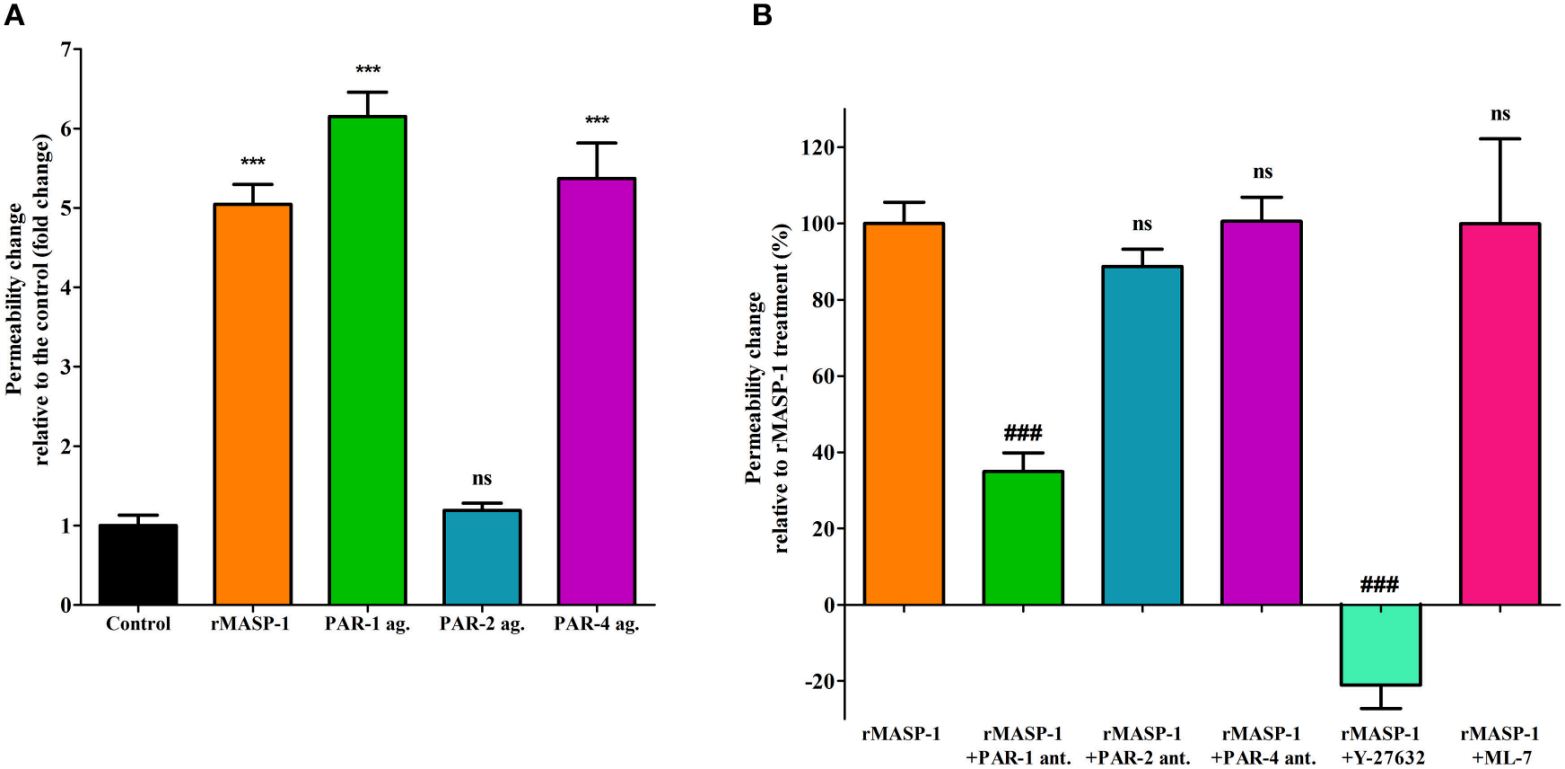
PAR-1 and ROCK plays a key role in the rMASP-1 induced endothelial permeability. Confluent layers of HUVECs were seeded onto 96 well plates pre-coated with biotinylated gelatin and were cultured for 2 days. Following cell treatment, streptavidin-Alexa488 was added to each well and after cell fixation, pictures were taken using fluorescence microscopy using an Olympus IX-81 fluorescence microscope and an Olympus XM-10 camera with 10x magnification Olympus lenses (numerical aperture: 0.3). Size of the stained area was determined on each image using the CellP software in three independent experiments. **(A)** Cells were treated with 2 μM rMASP-1 or PAR-agonists (4 μM PAR-1 agonist, 0.4 μM PAR-2 agonist or 1 mM PAR-4 agonist) or the culture medium alone for 20 min. Values are expressed as fold-change relative to the control. **(B)** Cells were pretreated with or without PAR antagonists (0.68 μM PAR-1 antagonist; 20 μM PAR-2 antagonist or 0.28 μM PAR-4 antagonist) for 10 min then were treated with 2 μM rMASP-1 for 20 min. Values are expressed as the percentage of rMASP-1 treatment (0% = cells treated only with the culture medium). ***p < 0.005, compared to the control; ###*p* < 0.005, compared to rMASP-1 treated; ns, non-significant.

Next, we investigated the PAR-dependence of the permeability increasing effect of rMASP-1. Similarly to rMASP-1, the PAR-1, and PAR-4 agonists significantly increased endothelial permeability, while the PAR-2 agonist did not (Figure 4A). Pretreating HUVECs with PAR-1 antagonist strongly blocked the rMASP-1 triggered permeability change, while antagonists of PAR-2 and PAR-4 were ineffective in the prevention of the rMASP-1 elicited permeability response (Figure 4B).

Then, we examined the role of MLCK and ROCK (two protein kinases important in cytoskeletal remodeling) in the permeability increasing effect of rMASP-1. ROCK inhibitor Y-27632 completely prevented the rMASP-1 induced permeability, and what is more, the intensity of paracellular transport through Y-27632 pretreated monolayers was even lower than that of the control. MLCK inhibitor, on the other hand, failed to prevent the effect of rMASP-1 (Figure 4B).

### rMASP-1 Induces MLC Phosphorylation and Rearrangement of the Actin Cytoskeleton in a PAR-1 and ROCK Dependent Manner

**Figure 5:**
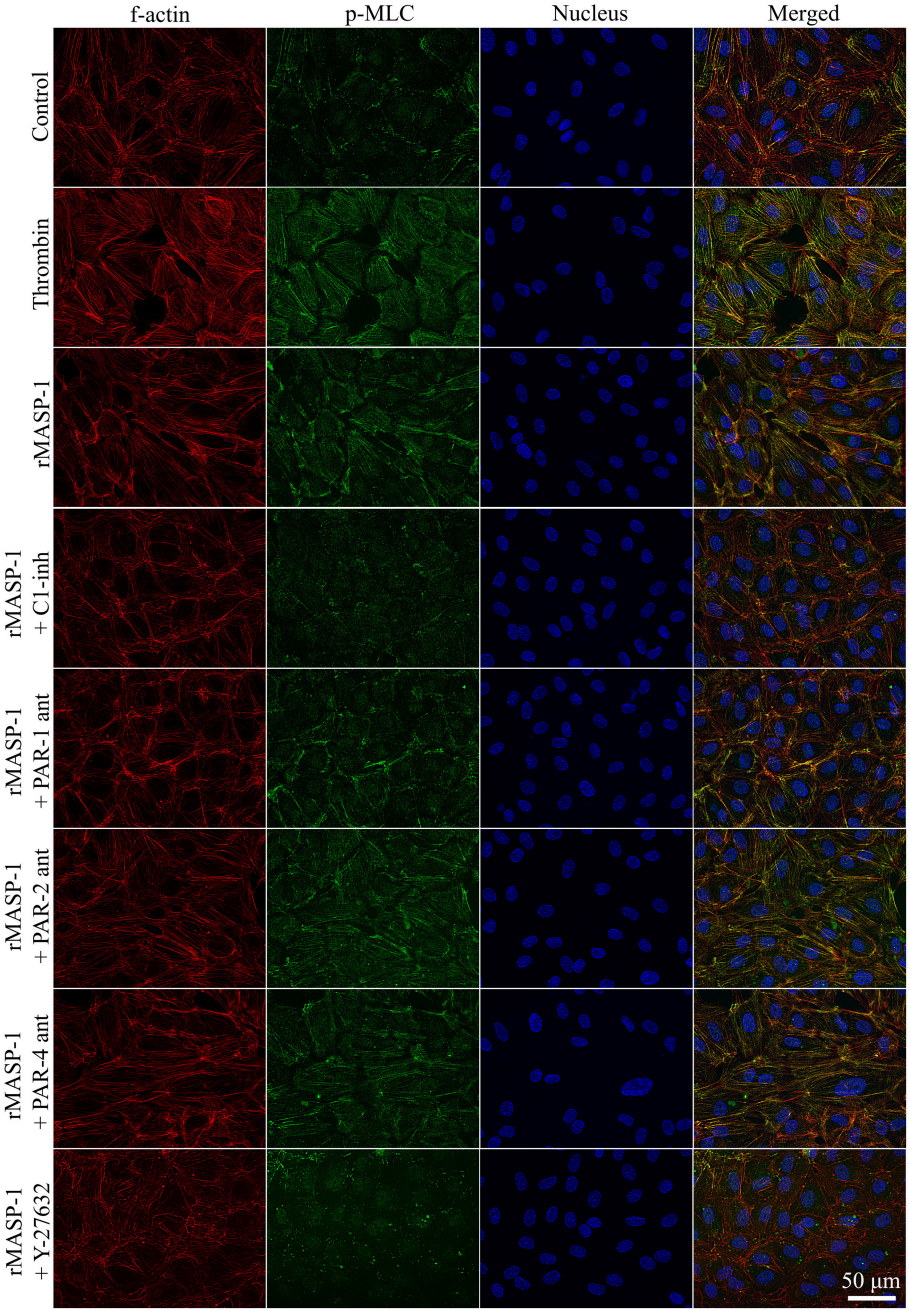
rMASP-1 treatment reorganizes the actin cytoskeleton of endothelial cells. Confluent layers of HUVECs were seeded onto 18 well ibidi™ slides and were cultured for 2 days. Cells were pretreated with or without PAR antagonists (0.68 μM PAR-1 antagonist; 20 μM PAR-2 antagonist or 0.28 μM PAR-4 antagonist) for 10 min or 2.5 μM ROCK inhibitor (Y-27632) for 15 min, then were treated with 2 μM rMASP-1 or 300 nM thrombin for 20 min. Cells treated with culture medium only served as a negative control. After fixation, cells were stained with anti-pMLC antibody. F-actin cytoskeleton was stained with phalloidin-Alexa488, cell nuclei were labeled with Hoechst 33258 and pictures were taken using an Olympus IX-81 fluorescence microscope and an Olympus XM-10 camera with 40x magnification Olympus lenses (numerical aperture: 0.75). Representative images from three independent experiments are shown. The scale bar applies to all photomicrographs.

We investigated the cytoskeletal changes underlying rMASP-1 induced endothelial permeability. Similarly to thrombin, rMASP-1 induced a strong MLC di-phosphorylation and rearrangement of the actin cytoskeleton of HUVECs resulting in stress fiber formation, moreover, di-phosphorylated MLC and rearranged actin fibers strongly co-localized (Pearson's *R* = 0.48). These effects of rMASP-1 were completely blocked by C1-INH (Figure 5) and SGMI-1 (data not shown). Pretreating the cells with PAR-1 antagonist also reduced the extent of MLC di-phosphorylation and stress fiber formation triggered by rMASP-1, while the PAR-2 and PAR-4 antagonists were ineffective. The ROCK inhibitor Y-27632 completely prevented the rMASP-1 induced cytoskeletal changes, moreover, this pretreatment resulted in a total absence of di-phosphorylated MLC and actin stress fibers (Figure 5). On the contrary, MLCK inhibitor ML-7 failed to avert the effect of rMASP-1.

### rMASP-1 Changes the Pattern of Molecules Important in Endothelial Cell Adhesion

**Figure 6:**
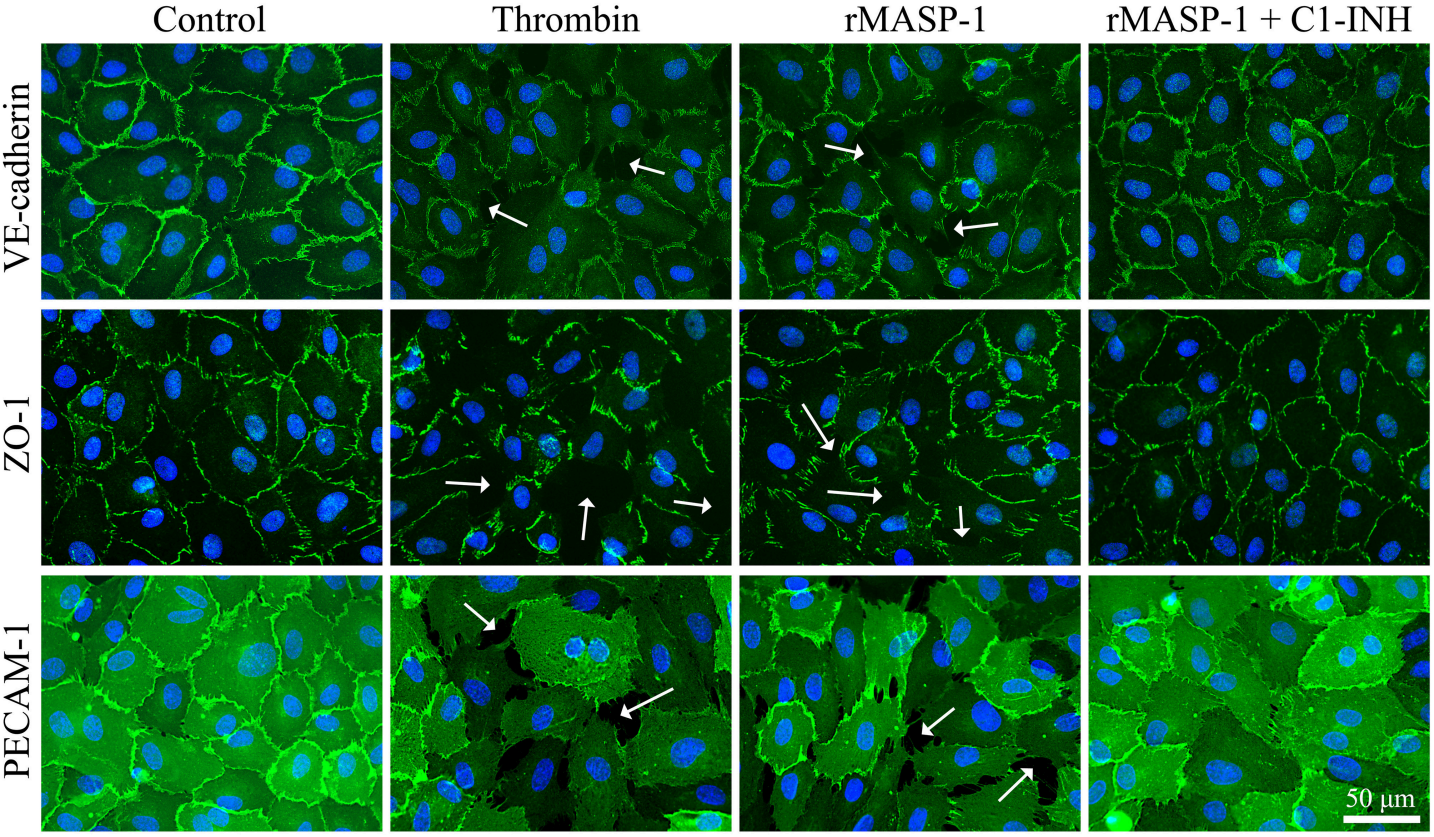
rMASP-1 treatment changes the pattern of endothelial cell adhesion molecules. Confluent layers of HUVECs were seeded onto 18 well ibidi™ slides and cultured for 2 days. Cells were either treated with 300 nM thrombin, 2 μM rMASP-1, a mixture of rMASP-1 and C1-inhibitor or with the culture medium alone (control) for 20 min. After fixation, cells were stained with anti-VE-cadherin, anti-ZO-1, or anti PECAM-1 antibodies (green). Cell nuclei were labeled with Hoechst 33258 (blue) and images were taken using an Olympus IX-81 fluorescence microscope and an Olympus XM-10 camera with 40x magnification Olympus lenses (numerical aperture: 0.75). Representative images from three independent experiments are shown. The scale bar applies to all photomicrographs. In the case of adhesion molecule VE-cadherin and intracellular adaptor ZO-1, the untreated control shows a well-organized, uninterrupted network of junctional molecules in the cell-cell junction areas, while rMASP-1 treatment (similarly to the positive control thrombin) resulted in a prominent disruption of this pattern (white arrows) indicating paracellular gap formation. This effect of rMASP-1 was completely blocked by C1-INH. A clear visualization of paracellular gaps can be seen in the case of adhesion molecule PECAM-1 as it is distributed evenly throughout the cell surface. Untreated cells form a continuous layer, while in response to rMASP-1 treatment, cells moved apart from each other to form paracellular gaps (white arrows), which was completely inhibited by C1-INH.

We also examined the rMASP-1 induced changes in the pattern of some important EC adhesion molecules. In these experiments, the untreated controls showed a well-organized, uninterrupted network of adhesion molecules VE-cadherin and PECAM-1, and intracellular adaptor molecule ZO-1, while rMASP-1 treatment resulted in a prominent disruption of this pattern and induced paracellular gap formation. This effect of rMASP-1 was completely blocked by C1-INH (Figure 6).

### rMASP-1 Significantly Changes the Expression of Several Permeability Related Genes and May Facilitate the Effect of Bradykinin

Finally, we performed whole transcriptome microarray analysis to investigate whether rMASP-1 influences the expression of permeability related genes in HUVECs as a prolonged effect. Treating the cells with rMASP-1 for 2 h eventuated in significant changes in the expression levels of 25 permeability related genes. For 12 out of these genes (9 up-regulated and 3 down-regulated) we found direct experimental evidence in the current literature verifying their role in regulating endothelial permeability (Table 1). For example, rMASP-1 up-regulated the expression of bradykinin B2 receptor (B2R) that is the endothelial cell surface receptor for the well-known edematogenic factor bradykinin. Furthermore, among the MASP-1 regulated genes, we identified an additional set of 6 genes (3 up-regulated and 3 down-regulated) with indirect experimental evidence—namely evidence for their role in modulating the effects of known regulators of the barrier properties of the endothelium, and also found a set of 7 genes (4 up-regulated and 3 down-regulated) that are suspected to affect endothelial permeability, but lack experimental evidence on their permeability-related role in EC systems (Supplemental Table 1). We also tested the effects of various known permeability increasing agonists such as LPS, histamine, thrombin and TNF-α on the gene expression of HUVECs, and found that expression of 68% (17 genes) of the permeability related genes regulated by rMASP-1 was also modulated by one or more of these mediators, while 8 genes were found to be regulated exclusively by rMASP-1 (Table 1 and Supplemental Table 1). Interestingly, with only one single exception, genes regulated both by rMASP-1 as well as by any of the above mentioned permeability increasing agonists manifested expression level alteration in the same direction regardless of the agonist applied. Furthermore, expression of 4 rMASP-1 regulated genes were also regulated by all of the above mentioned agonists.

## Discussion

Here we report the first case when the permeability increasing function of a complement serine protease is demonstrated. We provided evidence that MASP-1, a key serine protease of complement lectin pathway, induces endothelial hyperpermeability in a PAR-1 and ROCK dependent manner. Using whole-transcriptome analysis, we also showed that MASP-1 may have a prolonged effect on endothelial paracellular transport, as rMASP-1 treatment significantly affected the expression level of numerous permeability-related genes.

*In vivo*, MASP-1 binds to PRMs, but purification of these complexes are still quite challenging, moreover, the distribution of MASP-1 amongst the various PRMs remains largely unknown. In the current study, a recombinant catalytic fragment of MASP-1 (rMASP-1) was used, which contains only the three C-terminal domains of the native protein, therefore cannot dimerize or bind to pattern recognition molecules, but preserves enzymatic activity. This choice is justified by our earlier results, strongly suggesting that the catalytic fragment is necessary and sufficient for the cellular effects of the native MASP-1. First, the N-terminal fragment and the zymogen mutant forms of MASP-1 lacking enzymatic activity failed to induce intracellular Ca^2+^ mobilization in HUVECs, while the recombinant, catalytic fragment triggered a similar response as the native, MBL-complexed form (13). Furthermore, as we show in the current paper, C1-INH and the highly specific small molecule MASP-1 inhibitor SGMI-1 could completely prevent all the tested cellular effects of MASP-1.Taken together, although minor differences in their effects might occur between the recombinant, catalytic fragment of MASP-1 and the physiological MASP-1 complexes, the utilization of rMASP-1 may be a good model of most *in vivo* situations.

One of the main physiological roles of ECs is to form a tightly regulated barrier between the lumen of vessels and the interstitial space. This is achieved by a resting phenotype—generally characterized by well-oganized cell-cell junctions. However, various factors are able to transform this state into a leaky or hyperpermeable phenotype, when endothelial cell-cell junctions are disrupted and the endothelium becomes rich in paracellular gaps, facilitating paracellular transport. The xCELLigence system is an ideal platform for real-time screening of potential permeability increasing agents (47). With this system, we observed that, similarly to the well-known permeability increasing agonist thrombin, rMASP-1 induced a prominent decrease in the impedance of the HUVEC monolayer, strongly suggesting that rMASP-1 is able to increase endothelial permeability at least regarding the ion-current. To confirm this effect at the level of macromolecular transport, we utilized the XPerT permeability assay (28). XPerT is able to measure macromolecular transport similarly to Transwell™, however, the statistical reliablity of XPerT is superior due to its high-throughput testing capacity (28). In the XPerT system, rMASP-1 significantly increased the intensity of paracellular transport that could be blocked by C1-inhibitor as well as the highly specific MASP-1 inhibitor, SGMI-1.

Along the multistep, permeability increasing agonist mediated transition between the resting and the hyperpermeable phenotype of ECs the first step is their receptor-mediated activation, which is usually accompanied by a transient increase of intracellular [Ca^2+^] (22). For example, thrombin induced EC activation starts with a Ca^2+^ response initiated by the cleavage of EC surface PAR-1 (48). In our previous work, we have provided evidence that rMASP-1 elicits intracellular Ca^2+^ mobilization and thereby activates ECs (12, 13). We supposed that this activation is PAR-mediated, as we found that the catalytically inactive form of MASP-1 was unable to initiate any cellular response (13). Additionally, we have also shown earlier that rMASP-1 is indeed able to cleave PAR-1, PAR-2 and PAR-4 mimicking synthetic substrates—most efficiently the PAR-4 peptide, and PAR-4 was found to be important in MASP-1 triggered inflammatory response in HUVECs (12). In the current study, we investigated the PAR-dependence of rMASP-1 triggered EC activation and identified PAR-1 as the main mediator of this process as only the PAR-1 antagonist pretreatment could prevent the effects of rMASP-1. Nevertheless, PAR-1 antagonist could not completely block the permeability increasing and the Ca^2+^ mobilizing effects of rMASP-1, therefore, to a lesser extent, these may also be influenced by signaling via PAR-4, PAR heterodimers or other biased PAR signaling (49). A similar contribution of PAR-1 to the permeability increasing effect of thrombin is well-established in the literature (50–53). The differential contribution of PAR-1 and PAR-4 to the permeability and inflammatory response, respectively, shows that MASP-1 may regulate EC phenotype in a complex way.

Cytoskeletal rearrangement is an important prerequisite for paracellular gap formation. In their basal, quiescent state ECs show a cortical, ring-like organization of cytoskeletal F-actin. Permeability increasing agonists, such as thrombin, reorganize this structure into fiber-like bundles, called stress fibers, spreading throughout the cytoplasm. This transformation requires a Thr-18/Ser-19 diphosphorylated state of MLC established by phosphorylation and dephosphorylation events. Phosphorylation of MLC is catalyzed by MLCK and ROCK, while dephosporylation is mediated by MLC phosphatase (MLCP) (54). As ROCK can directly phosphorylate MLC, and also inactivate MLCP, it is considered a key regulator of cytoskeletal changes (55, 56). ROCK was found to mediate the endothelial barrier disrupting effect of thrombin as its selective inhibitor (Y-27632) prevented thrombin-induced MLC phosphorylation, stress fiber formation, and hyperpermeability in ECs (53). In our experiments, similarly to thrombin, rMASP-1 induced MLC diphosphorylation, and stress fiber formation. Pretreating ECs with Y-27632 blocked these effects, and also prevented rMASP-1 triggered endothelial hyperpermeability, while MLCK inhibitor ML-7 failed to prevent these events. Taken together, our results clearly show the key importance of ROCK in rMASP-1 elicited cytoskeletal changes and endothelial hyperpermeability, whereas the role of MLCK in this process needs to be further investigated.

Paracellular gap formation also requires the disruption of endothelial cell-cell junctions. To investigate the effects of rMASP-1 on these structures, we visualized three key molecules involved in the interconnection of ECs. Similarly to thrombin, rMASP-1 changed the pattern of molecules involved in EC-EC adhesion—it markedly disintegrated the network of VE-cadherin [which is a well-known indicator of endothelial hyperpermeability (57)], and also induced the disappearance of the tight junction component ZO-1 from the junctional areas. Furthermore, with the staining of PECAM-1 we provide evidence that—like thrombin—rMASP-1 increases the number and size of paracellular gaps, which is in line with the observed increase in paracellular permeability.

We have recently reported that in ECs, rMASP-1 regulates the expression of genes involved in the inflammatory reaction (29). Here we show that rMASP-1 treatment significantly changed the expression of 25 permeability-related genes. The majority of rMASP-1 regulated genes were also regulated by one or more of the four well-known permeability increasing agonists, LPS, histamine, thrombin and TNF-α. These agonists modulated EC gene expression in the same direction as rMASP-1. One of the most interesting findings of our transcriptome analysis was that rMASP-1 treatment significantly up-regulated the expression of bradykinin receptor B2R, which mediates the edematogenic effect of bradykinin. Bradykinin—proteolytically produced mainly by kallikrein—is known as the key edematogenic factor in the pathomechanism of HAE (39). However, we have recently shown that MASP-1 can also produce bradykinin by cleaving high-molecular-weight kininogen (11). Regardless of the source of bradykinin, up-regulation of B2R by MASP-1 can be important in the senzitization of ECs toward hyperpermeability.

Recently, the group of Wang et al., showed that a complement activation fragment, C4a serves as an untethered ligand for PAR-1 and PAR-4, and activates these receptors in a non-proteolytic manner (58). It is noteable that MASP-1 also contributes to the generation of C4a via MASP-2 activation. C4a mediated activation of PAR-1 also resulted in stress fiber formation, and increased endothelial permeability (58). These results underline the importance of our findings, and raise the possibility that components of the complement system may also affect the barrier function of the vessel wall.

According to our results, MASP-1 may play a role in several diseases, where edema formation is preceded by lectin pathway activation. One such condition can develop in patients with HAE, a life-threatening disease caused by the deficiency of C1-INH, the natural inhibitor of MASP-1, where MASP-1—due to its permeability increasing effect—may act as a potential triggering factor of edematous attacks. This is supported by the observation that infections can trigger HAE attacks (59) and by the recent finding that the levels of MASP-1 and MASP-1/C1-INH complexes are linked to disease severity in HAE patients (60). Furthermore, it is plausible that the permeability increasing effect of MASP-1 can contribute to the edema formation in septic or ischemia/reperfusion injury patients. In the future, *in vivo* experiments are needed to investigate the role of MASP-1 in the pathomechanism of the above mentioned diseases. If MASP-1 proves to be an indeed important player in HAE, sepsis, and ischemia/reperfusion, it can be a promising target for drug development.

## Ethics Statement

This study was carried out in accordance with the recommendations of WMA Declaration of Helsinki with written informed consent from all subjects. All subjects gave written informed consent in accordance with the Declaration of Helsinki. The protocol was approved by the Semmelweis University Institutional Review Board (permission number: TUKEB141/2015).

## Author Contributions

MLD designed and performed experiments, analyzed data, and wrote the manuscript. ZN performed the bioinformatics analysis. EK, ES, and VM contributed to cellular experiments and data analysis and commented on the manuscript. FW performed the xCELLigence experiments. ZD performed the transcriptome microarray experiments. AM and MAD designed experiments and commented on the manuscript. JR contributed to the maintenance and quality assurance of HUVEC cultures. GP, PG, and JD contributed to conceive the project, produced and purified recombinant MASP-1 and SGMI-1, and commented on the manuscript. LC conceived and supervised the project, designed experiments, analyzed data and edited the manuscript.

### Conflict of Interest Statement

The authors declare that the research was conducted in the absence of any commercial or financial relationships that could be construed as a potential conflict of interest.
