# Supplementary material for: MASP-1 Increases Endothelial Permeability
Source: Front Immunol. 2019 May 3;10:991. doi: 10.3389/fimmu.2019.00991 (PMC6509239; doi:10.3389/fimmu.2019.00991)
Supplement: Supplementary file 1 [file Table_1.docx]

| **Supplemental Table 1.** rMASP-1 significantly changes the expression of permeability related genes | | | | | | | | |
| --- | --- | --- | --- | --- | --- | --- | --- | --- |
| Median of fold change by rMASP-1 | Gene symbol | Gene name by HUGO Gene Nomenclature Committee (HGNC) | Description of the permeability related function | Type of evidence for a role in the regulation of endothelial permeability | Other activators | | | |
|  |  |  |  |  | LPS | Histamine | Thrombin | TNF-α |
| **Up-regulated** | | | | | | | | |
| 4.09 | F3 | coagulation factor III, tissue factor | Important mediator of the endothelial hyperpermeability induced by TNF-α, which exerts its permeability increasing effect by the up-regulation of endothelial cell surface tissue factor (Friedl et al. 2002). | Direct evidence | ↑ | ↑ | ↑ | ↑ |
| 3.38 | FZD7 | frizzled class receptor 7 | Adherent junction (AJ) protein which colocalizes with VE-cadherin and has an important role in the stabilization of the endothelial barrier (Ferreira Tojais et al. 2014). | Direct evidence |  |  |  |  |
| 3.35 | RCAN1 | regulator of calcineurin 1 | Regulator of calcineurin. Synthetized in response to histamine treatment, and has a role in reducing endothelial barrier breakdown (Ballesteros-Martinez et al. 2017). | Direct evidence | ↑ | ↑ | ↑ | ↑ |
| 2.78 | GHSR | growth hormone secretagogue receptor | Mediates the effect of ghrelin. Reduces the permeability increasing effect of LPS (Kwan et al. 2010) and also prevents the increase in the blood-brain barrier permeability following traumatic brain injury (Lopez et al. 2012). | Direct evidence | ↑ |  |  |  |
| 2.54 | FOXF1 | forkhead box F1 | Transcription factor important in the maintenance of endothelial barrier function (Cai et al. 2016). | Direct evidence | ↑ | ↑ | ↑ | ↑ |
| 2.32 | TLR2 | toll like receptor 2 | Activation of this receptor is required for postischemic permeability increase (Khandoga et al. 2009). | Direct evidence | ↑ |  |  |  |
| 2.24 | KITLG | KIT ligand | Binds to endothelial c-Kit receptor and increases endothelial permeability through the stimulation of VE-cadherin internalization (Kim et al. 2014; Im et al. 2016). | Direct evidence | ↑ | ↑ |  |  |
| 2.17 | BDKRB2 | bradykinin receptor B2 | Receptor for the well-known edematogenic factor bradykinin. Also an important pharmacological target in HAE therapy (Farkas 2016). | Direct evidence |  |  | ↑ | ↑ |
| 2.03 | TGFBR1 | transforming growth factor beta receptor 1 | TGF-β induces endothelial cell contraction and increases endothelial permeability through this receptor (Birukova et al. 2005; Antonov et al. 2012). | Direct evidence | ↑ | ↑ | ↑ |  |
| 3.39 | SNAI2 | snail family transcriptional repressor 2 | Transcription factor, also up-regulated upon VEGF treatment in HUVECs. Knocking out SNAI2 prevented the VEGF induced reduction in the mRNA level of Claudin-5, a key component of tight junctions (TJs) (Laakkonen et al. 2017). | Indirect evidence |  |  |  |  |
| 2.94 | SMAD6 | SMAD family member 6 | Signaling mediator which inhibits TGF-β1 signaling through TGFβ receptor 1 (Imamura et al. 1997). | Indirect evidence |  |  | ↑ | ↓ |
| 2.39 | VAV3 | vav guanine nucleotide exchange factor 3 | Guanine nucleotide exchange factor for Rho family GTP‑ases. Involved in ephrin-A1/ephrin-A2 receptor signaling (Hunter et al. 2006), which leads to increased endothelial permeability (Larson et al. 2008). | Indirect evidence | ↑ |  |  |  |
| 2.98 | MPP7 | membrane palmitoylated protein 7 | Important adapter protein involved in the maintenance of the dynamic stability of epithelial TJs (Stucke et al. 2007). | Potential role | ↑ | ↑ |  |  |
| 2.47 | NUAK2 | NUAK family kinase 2 | The only kinase up-regulated by TNF-α in ECs. Phosphorylates MYPT1 subunit of MLCP(Yamamoto et al. 2008), and may be involved in the conversion of F-actin to G-actin. Overexpression causes cell detachment from the substrate (Suzuki et al. 2003). | Potential role | ↑ |  | ↑ | ↑ |
| 2.25 | PNN | pinin, desmosome associated protein | Nuclear phosphoprotein regulating cell-cell adhesion. Knocking down of PNN resulted in the loss of epithelial cell-cell adhesion (Joo et al. 2005). | Potential role | ↑ |  |  |  |
| 2.11 | STAB2 | stabilin 2 | Important systemic scavenger receptor for hyaluronan and heparin, and extracellular matrix components (such as chondroitin sulfate and dermatan sulfate) which are known to influence endothelial permeability (Harris and Weigel 2008). | Potential role |  |  |  |  |
| **Down-regulated** | | | | | | | | |
| -2.90 | EDNRB | endothelin receptor type B | Receptor for endothelin-1, which reduces the permeability increasing effect of bradykinin and ATP through this receptor (Victorino et al. 2004). | Direct evidence | ↓ | ↓ | ↓ | ↓ |
| -2.58 | APLNR | apelin receptor | Mediator of the effects of apelin, which is important for the stabilization of the endothelial barrier of both blood and lymphatic vessels (Sawane et al. 2011; Sawane et al. 2013; Tatin et al. 2017). | Direct evidence |  |  |  |  |
| -2.30 | CCR3 | C-C motif chemokine receptor 3 | Eotaxin increases endothelial permeability through this receptor (Jamaluddin et al. 2009). | Direct evidence | ↓ |  |  |  |
| -2.39 | CAMSAP3 | calmodulin regulated spectrin associated protein family member 3 | Binds minus ends of microtubules to zonula adherens, thereby stabilizing AJs (Meng et al. 2008). | Indirect evidence |  |  |  |  |
| -2.11 | P2RY12 | purinergic receptor P2Y12 | Purinergic receptor, stimulation of which leads to a reduced level of intracellular cAMP, which is known to contribute to the disruption of the endothelial barrier (Simon et al. 2002). A P2Y12 receptor antagonist reduced endothelial dysfunction in patients with a history of acute coronary syndrome (Torngren et al. 2013). | Indirect evidence |  |  |  |  |
| -2.00 | PRKCG | protein kinase C gamma | May have a role in the permeability increase occurring after hypoxia-reoxigenation(Fleegal et al. 2005). Also plays a role in thrombin induced ezrin/radixin/moesin and myosin light chain phosphorylation, which leads to increased permeability(Adyshev et al. 2013). | Indirect evidence |  |  |  |  |
| -3.72 | CLDN22 | claudin 22 | May be an important transmembrane constituent of endothelial TJs(Ohtsuki et al. 2008). | Potential role | ↓ | ↓ | ↓ |  |
| -2.18 | LBP | lipopolysaccharide binding protein | Plasma protein that binds LPS, which is necessary for the LPS induced activation of ECs(Pugin et al. 1993). | Potential role |  |  |  |  |
| -2.13 | MARVELD3 | MARVEL domain containing 3 | Transmembrane TJ protein of the occluding family. Important in the maintenance of epithelial barrier function(Steed et al. 2009). | Potential role | ↓ |  |  |  |
| Terms and pathways used to filter out possible permeability related genes:  **GO** - permeability; junction; actin; adhesion; camp; cgmp; rho; calcium  **REACTOME**- Cell junction organization; Extracellular matrix organization; Cell surface interactions at the vascular wall; Toll-like receptors cascades; Complement cascade; Nucleotide-binding domain, leucine rich repeat containing receptor (NLR) signaling pathways  **KEGG** - Cell adhesion molecules; Adherens junction; Focal adhesion; Tight junction; Gap junction; Complement and coagulation cascades; Toll-like receptor signaling pathway; NOD-like receptor signaling pathway; RIG-I-like receptor signaling pathway; Leukocyte transendothelial migration | | | | | | | | |

**Supplemental Table 1**: rMASP-1 significantly changes the expression of permeability related genes.

Confluent layers of HUVECs from four individuals were cultured in 6 well plates and treated for 2 hours with 0.6 μM rMASP-1. To compare the effects of rMASP‑1 with other endothelial cell (EC) activators, cells were treated with 300 nM thrombin, 10 ng/mL TNFα, 100 ng/mL LPS, or 50 μM histamine. From the set of genes significantly changed by rMASP-1, possible permeability-related genes were filtered out using REACTOME, KEGG and GO databases. Experimental evidence for the permeability related function of these genes were verified according to the current literature. Genes that could be linked to permeability regulation were divided into three groups based on the type of evidence for their role in the modulation of endothelial permeability: i) direct evidence – protein products of these genes have been directly proven to affect endothelial permeability ii) indirect evidence – protein products of these genes have been proven to affect the function of known permeability regulating factors, and iii) potential role – protein products of these genes are suspected to influence the barrier properties of the endothelium. Genes in the i) and ii) categories are also proven to be expressed in ECs at protein level. The table contains the median fold-change (FC) values of the 4 independent HUVECs.

**↑**: Gene expression was up-regulated by the given activator, **↓**: Gene expression was up-regulated by the given activator

**References for Supplemental Table 1.**

Adyshev, D. M., S. M. Dudek, et al. (2013). "Ezrin/radixin/moesin proteins differentially regulate endothelial hyperpermeability after thrombin." Am J Physiol Lung Cell Mol Physiol **305**(3): L240-255.

Antonov, A. S., G. N. Antonova, et al. (2012). "Regulation of endothelial barrier function by TGF-beta type I receptor ALK5: potential role of contractile mechanisms and heat shock protein 90." J Cell Physiol **227**(2): 759-771.

Ballesteros-Martinez, C., N. Mendez-Barbero, et al. (2017). "Endothelial Regulator of Calcineurin 1 Promotes Barrier Integrity and Modulates Histamine-Induced Barrier Dysfunction in Anaphylaxis." Front Immunol **8**: 1323.

Birukova, A. A., D. Adyshev, et al. (2005). "ALK5 and Smad4 are involved in TGF-beta1-induced pulmonary endothelial permeability." FEBS Lett **579**(18): 4031-4037.

Cai, Y., C. Bolte, et al. (2016). "FOXF1 maintains endothelial barrier function and prevents edema after lung injury." Sci Signal **9**(424): ra40.

Farkas, H. (2016). "Icatibant as acute treatment for hereditary angioedema in adults." Expert Rev Clin Pharmacol **9**(6): 779-788.

Ferreira Tojais, N., C. Peghaire, et al. (2014). "Frizzled7 controls vascular permeability through the Wnt-canonical pathway and cross-talk with endothelial cell junction complexes." Cardiovasc Res **103**(2): 291-303.

Fleegal, M. A., S. Hom, et al. (2005). "Activation of PKC modulates blood-brain barrier endothelial cell permeability changes induced by hypoxia and posthypoxic reoxygenation." Am J Physiol Heart Circ Physiol **289**(5): H2012-2019.

Friedl, J., M. Puhlmann, et al. (2002). "Induction of permeability across endothelial cell monolayers by tumor necrosis factor (TNF) occurs via a tissue factor-dependent mechanism: relationship between the procoagulant and permeability effects of TNF." Blood **100**(4): 1334-1339.

Harris, E. N. and P. H. Weigel (2008). "The ligand-binding profile of HARE: hyaluronan and chondroitin sulfates A, C, and D bind to overlapping sites distinct from the sites for heparin, acetylated low-density lipoprotein, dermatan sulfate, and CS-E." Glycobiology **18**(8): 638-648.

Hunter, S. G., G. Zhuang, et al. (2006). "Essential role of Vav family guanine nucleotide exchange factors in EphA receptor-mediated angiogenesis." Mol Cell Biol **26**(13): 4830-4842.

Im, J. E., S. H. Song, et al. (2016). "Src tyrosine kinase regulates the stem cell factor-induced breakdown of the blood-retinal barrier." Mol Vis **22**: 1213-1220.

Imamura, T., M. Takase, et al. (1997). "Smad6 inhibits signalling by the TGF-beta superfamily." Nature **389**(6651): 622-626.

Jamaluddin, M. S., X. Wang, et al. (2009). "Eotaxin increases monolayer permeability of human coronary artery endothelial cells." Arterioscler Thromb Vasc Biol **29**(12): 2146-2152.

Joo, J. H., R. Alpatov, et al. (2005). "Reduction of Pnn by RNAi induces loss of cell-cell adhesion between human corneal epithelial cells." Mol Vis **11**: 133-142.

Khandoga, A. G., A. Khandoga, et al. (2009). "Postischemic vascular permeability requires both TLR-2 and TLR-4, but only TLR-2 mediates the transendothelial migration of leukocytes." Shock **31**(6): 592-598.

Kim, J. Y., J. S. Choi, et al. (2014). "Stem cell factor is a potent endothelial permeability factor." Arterioscler Thromb Vasc Biol **34**(7): 1459-1467.

Kwan, R. O., E. Cureton, et al. (2010). "Ghrelin decreases microvascular leak during inflammation." J Trauma **68**(5): 1186-1191.

Laakkonen, J. P., J. P. Lappalainen, et al. (2017). "Differential regulation of angiogenic cellular processes and claudin-5 by histamine and VEGF via PI3K-signaling, transcription factor SNAI2 and interleukin-8." Angiogenesis **20**(1): 109-124.

Larson, J., S. Schomberg, et al. (2008). "Endothelial EphA receptor stimulation increases lung vascular permeability." Am J Physiol Lung Cell Mol Physiol **295**(3): L431-439.

Lopez, N. E., M. J. Krzyzaniak, et al. (2012). "Ghrelin prevents disruption of the blood-brain barrier after traumatic brain injury." J Neurotrauma **29**(2): 385-393.

Meng, W., Y. Mushika, et al. (2008). "Anchorage of microtubule minus ends to adherens junctions regulates epithelial cell-cell contacts." Cell **135**(5): 948-959.

Ohtsuki, S., H. Yamaguchi, et al. (2008). "mRNA expression levels of tight junction protein genes in mouse brain capillary endothelial cells highly purified by magnetic cell sorting." J Neurochem **104**(1): 147-154.

Pugin, J., C. C. Schurer-Maly, et al. (1993). "Lipopolysaccharide activation of human endothelial and epithelial cells is mediated by lipopolysaccharide-binding protein and soluble CD14." Proc Natl Acad Sci U S A **90**(7): 2744-2748.

Sawane, M., K. Kajiya, et al. (2013). "Apelin inhibits diet-induced obesity by enhancing lymphatic and blood vessel integrity." Diabetes **62**(6): 1970-1980.

Sawane, M., H. Kidoya, et al. (2011). "Apelin attenuates UVB-induced edema and inflammation by promoting vessel function." Am J Pathol **179**(6): 2691-2697.

Simon, J., A. K. Filippov, et al. (2002). "Characterization and channel coupling of the P2Y(12) nucleotide receptor of brain capillary endothelial cells." J Biol Chem **277**(35): 31390-31400.

Steed, E., N. T. Rodrigues, et al. (2009). "Identification of MarvelD3 as a tight junction-associated transmembrane protein of the occludin family." BMC Cell Biol **10**: 95.

Stucke, V. M., E. Timmerman, et al. (2007). "The MAGUK protein MPP7 binds to the polarity protein hDlg1 and facilitates epithelial tight junction formation." Mol Biol Cell **18**(5): 1744-1755.

Suzuki, A., G. Kusakai, et al. (2003). "Induction of cell-cell detachment during glucose starvation through F-actin conversion by SNARK, the fourth member of the AMP-activated protein kinase catalytic subunit family." Biochem Biophys Res Commun **311**(1): 156-161.

Tatin, F., E. Renaud-Gabardos, et al. (2017). "Apelin modulates pathological remodeling of lymphatic endothelium after myocardial infarction." JCI Insight **2**(12).

Torngren, K., J. Ohman, et al. (2013). "Ticagrelor improves peripheral arterial function in patients with a previous acute coronary syndrome." Cardiology **124**(4): 252-258.

Victorino, G. P., C. R. Newton, et al. (2004). "Endothelin-1 decreases microvessel permeability after endothelial activation." J Trauma **56**(4): 832-836.

Yamamoto, H., S. Takashima, et al. (2008). "Identification of a novel substrate for TNFalpha-induced kinase NUAK2." Biochem Biophys Res Commun **365**(3): 541-547.
